# Macrophages as prognostic biomarkers in human melanoma

**DOI:** 10.18632/oncoscience.442

**Published:** 2018-06-30

**Authors:** Rafael Samaniego, Alba Gutiérrez-Seijo, Paloma Sánchez-Mateos

**Affiliations:** Unidad de Microscopía Confocal, Instituto de Investigación Sanitaria Gregorio Marañón, Madrid, Spain.

**Keywords:** melanoma, prognostic factor, macrophages, CCL20/CCR6, protumoral phenotype

Chemokines produced at the tumor micro-environment can be exploited by tumor cells to proliferate and invade, or may promote tumor growth indirectly by acting on stromal cells. CCR6/CCL20 is a relevant chemotactic axis involved in the progression and dissemination of different human cancers [1]. We have recently shown the existence of a previously unnoticed paracrine loop between stromal CCL20 and tumor CCR6 in primary cutaneous melanoma [2], different to the autocrine loop reported in epithelial tumors [1]. CCL20 expression was largely restricted to the stroma of primary melanomas that eventually developed metastasis, and proved to be a Breslow-independent prognostic factor associated with distant metastasis and worse patient survival. By contrast, we did not find any correlation between tumor CCR6 expression and metastatic dissemination [2].

Among stromal cells, tumor-associated macrophages (TAMs) were clearly identified as the major producers of CCL20 in metastatic primary melanomas [2]. This selective production most probably reflects the capacity of TAMs to adopt distinct activation states or to reprogram their phenotype in response to environmental changes occurring along tumor initiation, progression and dissemination. Several studies in cancer mouse models have shown that TAMs adopt a temporally and spatially varying phenotypic spectrum between the inflammatory/M1 (MHC-II^hi^ CD11c^hi^) and alternative/M2 (MHC-II^lo^ CD11c^lo^) polarization extremes [3], whereas reports identifying macrophage subtypes in human cancers are scarce. We selected the proteins CD11c and CD209 as potential polarization markers to discriminate subpopulations of TAMs by multicolor confocal single-cell analysis in human melanoma frozen-tissues. We found that the phenotype of melanoma TAMs (CD14^+^ CD115^+^ CD1c^−^ CD141^−^) oscillated between two clearly differentiated subsets, CD11c^hi^ CD209^lo^ CD163^lo^ CD163L1^lo^ and CD11c^lo^ CD209^hi^ CD163^hi^ CD163L1^hi^ [2], being the last three proteins commonly used as M2-like markers. Interestingly, a similar phenotypic dichotomy has been observed in first-trimester placental and inflammatory bowel disease tissues using CD11c/CD209 as inverse markers. In these studies, CD11c^hi^ CD209^lo^ decidual and lamina-propria macrophages were associated with an inflammatory phenotype, whereas CD11c^lo^ CD209^hi^ cells displayed profiles more similar to tissue-remodeling and tissue-resident macrophages, respectively [4, 5].

Remarkably, the two opposite TAMs subsets defined by CD11c/CD209 were found in all primary melanomas, independently of whether they were metastatic or non-metastatic during patient follow-up. By contrast, CCL20 was produced by most TAMs in metastatic primary melanomas, independently of other phenotype or subset distinction. Consistently, co-culture assays with melanoma cells induced the production of the chemokine in monocyte-derived macrophages differentiated with either GM-CSF (M1-like) or M-CSF (M2-like) [2]. Importantly, TNF and VEGF-A proteins, which play known tumor-promoting roles [3], were also preferentially produced by TAMs from metastatic primary melanomas [2], independently of the polarization state of macrophages. In line with our results, the tumor-supporting transcriptional profile of blood monocytes from renal cell carcinoma patients included upregulated levels of CCL20, TNF and VEGF-A [6].

**Figure 1:**
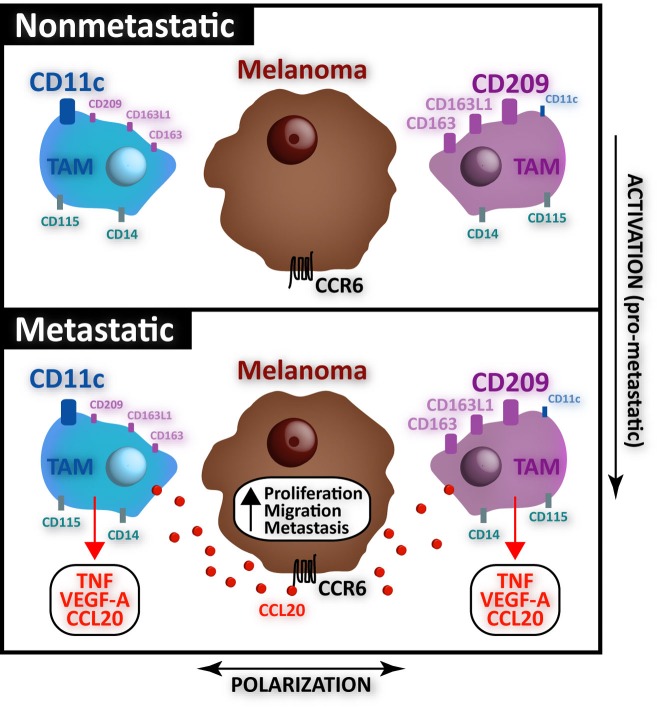
Schematic representation of the different polarization and activation states of melanoma TAM

Altogether our results suggest that in primary human melanoma, pro-metastasic TAMs might originate as a result of a polarization-independent functional activation process (Figure 1). Our findings do not fit well with the prevalent idea that tumor evolution is associated with the accumulation of a particular polarization state, as shown in longitudinal studies of mouse cancers where a M1>M2 transition occurs during tumor progression [3, 7]. However, primary tumor growth and metastatic spreading are independent processes that may be independently associated with diverse TAM polarization/activation states. Translationally, identification of pro-metastatic TAM protein signatures, such as the one we report in melanoma (CCL20/TNF/VEGF-A), may be useful not only for developing macrophage-centered therapeutic approaches in which tumor-supporting TAMs are potential targets [7], but also as prognostic biomarkers that may help identify patients with higher risk of metastatic spreading in order to guide early treatments.
